# A New Electronic Monitoring Device to Measure Medication Adherence: Usability of the Helping Hand™

**DOI:** 10.3390/s100301535

**Published:** 2010-03-01

**Authors:** Leentje De Bleser, Birgit Vincke, Fabienne Dobbels, Mary Beth Happ, Bart Maes, Johan Vanhaecke, Sabina De Geest

**Affiliations:** 1 Centre for Health Services and Nursing Research, Katholieke Universiteit Leuven, Leuven, Kapucijnenvoer 35 box 7001, B-3000 Leuven, Belgium; E-Mails: leentje.debleser@med.kuleuven.be (L.D.B.); birgitvincke@gmail.com (B.V.); fabienne.dobbels@med.kuleuven.be (F.D.); 2 School of Nursing, University of Pittsburgh, 3500 Victoria Street, Pittsburgh, PA 15261, USA; E-Mail: mhapp@pitt.edu; 3 Heilig-Hart Ziekenhuis Roeselare-Menen, Wilgenstraat 2, Roeselare, Belgium; E-Mail: BMaes@hhr.be; 4 Heart Transplantation Program, University Hospitals of Leuven, Leuven, Belgium; E-Mail: johan.vanhaecke@uz.kuleuven.be; 5 Institute of Nursing Science, University of Basel, Basel, Switzerland

**Keywords:** electronic monitoring, usability, user performance, satisfaction, acceptability, mixed method design

## Abstract

The aim of this study was to test the *user performance, satisfaction* and *acceptability* of the Helping Hand™ (B&O Medicom) electronic medication adherence monitor. Using a mixed-method design, we studied 11 kidney transplant patients and 10 healthy volunteers during three weeks. Although testing showed positive usability aspects, several areas requiring technical improvement were identified: the most important obstacles to usability and acceptability were the weak sound signal, problems loading the medication, and the fact that only one medication could be used at a time.

## 1. Introduction

Non-adherence is a prevalent problem in chronically ill populations, which may result in poor clinical and economic outcomes [1]. Measurement of medication non-adherence is crucial to identify patients at risk for poor outcomes and to evaluate adherence-enhancing interventions in all clinical settings. Cross-validation studies show electronic monitoring (EM) to be the most sensitive method available for measuring medication non-adherence (NA) [2–4], providing uniquely time stamped data on medication adherence dynamics over time [5]. Numerous electronic monitoring devices to measure adherence exist. Some devices are developed to measure medication taking habits e.g., MEMS and Helping Hand™. Other devices are developed for home monitoring of the effects of medication e.g., blood pressure monitoring, spirometry. Elaborating on these devices would be interesting, although would lead us too far, given the scope of this article. Of the EM systems currently in use, the Medication Event Monitoring System (MEMS, Aardex, CH) is most popular. Yet, its drawbacks include confidentiality issues, as it uses a rather bulky pill bottle that may be visible to others during medication taking. The size of the pill bottle depends on the types of prescribed medications and the period of time until the next medical appointment. Also, a pill bottle is often impractical: some tablets have to stay in sealed blisters until ingestion, as exposure to moisture, air, light, or microbiological contamination can affect them adversely [6]. For use with MEMS, then, blister cards need to be cut apart by a pharmacist (to avoid damaging the blisters) to emulate individual pills [6], which is very time consuming.

**Figure 1.**
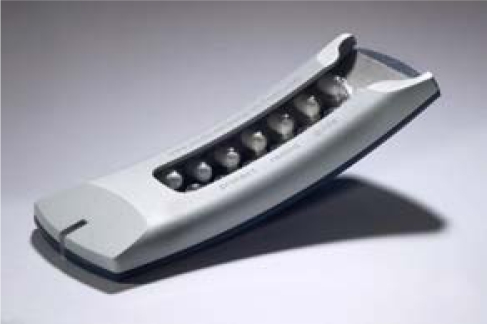
The Helping Hand™ (Bang & Olufsen).

Recently, the Helping Hand™ (HH) (see Figure 1) EM system was launched by Bang & Olufsen Medicom. As with the MEMS system, a processor chip monitors presumed tablet intake by registering the date and time of each use. The Helping Hand™ gives acoustic reminders at the time medication taking is prescribed. Then, patients move the blister out of the Helping Hand™, take their medication as prescribed and reinsert the blister. When reinserting the blister, NA feedback is given by a red/orange/green light system. For example if a patient is on a twice daily regimen, 14 doses need to be taken within one week: the green light means that 14 out of 14 doses are taken (100% adherence); the orange light indicates that 12 or 13 doses are taken (85.7%–92.9% adherence), and the red light represents that 11 doses or less are taken within one week (≤78.8% adherence). These stringent cut-off values are based on previous work showing that minor deviations from dosing schedule are associated with late acute rejections [7,8]. The cut-offs can be programmed differently depending on the disease population under study. In addition, the HH could generate data printouts that can be shown to patients to discuss adherence patterns.

The accuracy of the HH’s monitor is reported in another article (De Bleser *et al.* provisionally accepted for publication in Sensors), concluding that perfect functioning was observed in 70% to 87% of the HH. In addition to accuracy, its use from the patient’s perspective should also be assessed. These aspects of usability require subjective user-centered testing. The aim of the present study is to evaluate the usability of the HH in terms of user performance [9], satisfaction [10] and acceptability [11].

## 2. Methods

### 2.1. Design

A conceptual framework that can be used to evaluate the different dimensions of usability is described elsewhere (De Bleser *et al.*, work in progress). A combination of quantitative and qualitative descriptive methods (mixed methods) was used [12], employing a two phase concurrent triangulation strategy (for complementarity and completeness) [13]. In *Phase 1*, participants were first instructed on the device’s features and operation. The think aloud method [12] and a quantitative questionnaire were used (see ‘procedure’ below) to identify user performance aspects, after which each subject was provided with a device for daily use during three consecutive weeks. *Phase 2*, which began three weeks later, involved a semi-structured qualitative interview and quantitative survey questions using Likert scales to explore aspects of satisfaction and acceptability. The quantitative and qualitative data collections were conducted separately but had equal priority.

### 2.2. Sample and setting

Two subject samples were used: healthy volunteers and kidney transplant patients (KTx). KTx patients were selected for the study because it is known that strict medication adherence is of critical importance in this population [14]. Therefore, electronic monitoring devices, such as the Helping Hand™, may be very useful for this patient population. However, transplant patients’ experiences with the Helping Hand™ may be influenced by their medical condition. For instance, some immunosuppressive drugs can cause tremor or blurry vision. Therefore, healthy volunteers were included because they are not expected to have such challenges. All subjects were enrolled between September 2007 and December 2007. To select healthy volunteers and KTx patients, purposive criterion-related block sampling [12] was used to ensure a balanced distribution regarding age, gender and education because it is assumed that user experiences and medication taking are influenced by these factors [14]. Inclusion criteria were: age of 18 years or older, willingness to use the Helping Hand™ as directed for a three week period, and willingness to undergo the associated interviews. Healthy volunteers were recruited using a snowball technique. We first asked colleagues if they could nominate possible volunteers, who were contacted by telephone. Those providing oral consent were asked to nominate further candidates (e.g., colleagues, neighbors).

KTx patients were recruited from the Heilig-Hart Hospital, Roeselare-Menen, Belgium. In addition to the inclusion criteria that were used for the healthy volunteers, patients had to be first-time KTx recipients, more than 1 year post-transplant, and on twice-daily tacrolimus regimens. Exclusion criteria were: a history of retransplantation; multi-organ transplantation; and participation in a tacrolimus-related clinical trial or any study that could interfere with ours.

All subjects provided written informed consent. This study was approved by the ethics committee of the Heilig-Hart Hospital, Roeselare-Menen, Belgium (B11720072609).

### 2.3. Assessments and study protocol

#### 2.3.1. Demographic and clinical characteristics

Demographic characteristics (age, gender, educational level, and marital status) were self-reported during the initial interview, along with information about sight, hearing, and fine motor control. Patients provided information on transplantation date, follow-up frequencies, immunosuppressive regimens, tacrolimus dosages, and previous experience with electronic devices.

#### 2.3.2. The Helping Hand™ monitoring device

The Helping Hand™ device is a flat, slightly arched blister card holder. This resembles a telephone handset. The Helping Hand™ is 16 cm long, 6 cm wide and 1 cm thick and was provided to each participant. Subjects then had to load these with blister cards (appropriate tacrolimus doses (0.5, 1 or 5 mg) for KTx patients, placebos for healthy volunteers). Participants could program their own reminder times, but these had to represent a standard twice-daily regimen, *i.e.*, 12-hour intervals, and had to remain unchanged for the entire study period.

#### 2.3.3. Usability testing

Three usability dimensions were tested: user performance [9] in Phase 1; and satisfaction [10] and acceptability [11] in Phase 2.

##### Phase 1: User performance evaluation

Safe and effective medical device use relies mainly on three device-user interaction factors [9]: use environment (the mental and physical workloads involved in using the device [11]); user characteristics (users’ abilities and limitations regarding the device’s safe and effective use; coordination; cognitive ability; and memory [15]); and device-user interface characteristics (all components of the device with which users interact during use, preparation for use, or maintenance [16]). User performance was evaluated using three methods:

- Counting user errors: After being instructed about the critical steps to set up and operate the HH, participants were asked to handle the device. While participants were performing each concrete operational step (20 steps in total), the researcher used a structured assessment to observe the number of errors (see Table 2).
- Timing user tasks: While participants were operating the HH, the time required in seconds was registered. Three specific tasks were tested: initial activation; reprogramming (*i.e.*, setting the acoustic and visual reminders to the user’s preferences); and removal of a medication dose (see Table 2).
- Think-aloud sessions: During the use of the device, patients were asked to express their thoughts while performing the target task (think aloud). The think-aloud method was developed by Lewis [16] as a method used to gather data in usability testing focusing on how easy it is for new users to accomplish tasks associated with the device. Users are asked to say whatever they are looking at, thinking, doing and feeling, as they go through the set of specific tasks or sequence of actions when using a device. Observers objectively take notes of everything that users say, without attempting to interpret their actions and words. The purpose of the think-aloud technique is to make explicit what subjects’ thoughts and experiences when performing a specific task.

All sessions were audio-taped. The researcher activated the audio recorder, gave a standardized description of all critical steps in setting and operating the Helping Hand™, and explained the think-aloud method, which the subject first practiced, then used. To minimize the behavior of giving desirable answers, subjects were assured that the study was purely descriptive, that no wrong answers were possible, and that their medication taking behavior (*i.e.*, adherence or non-adherence) was not being evaluated. During every interview, the researcher noted her own remarks and observations, e.g., subject’s facial expression, interaction with other family members.

At the end of their sessions, subjects were asked to rate the difficulty of interacting with the device on a 10-point Likert scale (0 = not difficult at all; 10 = extremely difficult). After the think-aloud session, the subject was asked to use the device for three weeks, noting ease of use, any difficulties encountered, and any positive or negative impressions of the system. Before leaving, the interviewer scheduled the next visit. The researcher transcribed the session recording as soon as possible, including notes from observations and impressions.

##### Phase 2: Satisfaction and acceptability

Satisfaction is reported in terms on six dimensions [10]. The *physical dimension* involves the impact of the device’s physical characteristics on users or their home environments. The *privacy dimension* deals with how inconspicuously users can employ the device, e.g., in a work environment [17]. The *human interaction dimension* refers to the degree to which the device influences in-person interaction with health care providers. The *self-concept dimension* involves psychological consequences of the device’s use, including a sense of dependency on it. The affect of the device’s use on daily routines or rituals, e.g., replacing pillboxes, summarizes the *routine dimension*. Finally, the *dimension of sustainability* reflects users’ concerns about limitations on long-term use, including affordability or diminishing personal capacities. *Acceptability* indicates user opinions of whether they would incorporate the device into their adherence management routines [18], and if so, how much they would consider paying for it.

At the end of the 3-week device trial, user satisfaction and acceptability of the device were assessed via a semi-structured interview and a brief questionnaire. The semi-structured interview contained 12 questions, covering each dimension of satisfaction and acceptability. The researcher asked whether the subject had used the device throughout the monitoring period. If not, the reason was obtained. Interviews lasted approximately 20–25 minutes.

The questionnaire comprised 5 questions on satisfaction that could be answered on a 5-point Likert scale. To avoid forcing judgments, each item included an option to express ‘no opinion’ (“3” on the 5-point Likert scale). This questionnaire was based on one that was previously used to evaluate software investigating cancer patients’ quality of life [19]. In that study, the reliability coefficient was 0.91, indicating a high internal consistency. However, the questionnaire has not been used to evaluate satisfaction and acceptability in areas other than computer applications.

## 3. Data Analysis

### 

#### 

##### Phase 1: User performance evaluation

The median times for reprogramming and operating the device were calculated for all participants, as well as for patients and volunteers separately. Regarding the systematic breakdown of all steps involved in using the device, participants’ attempts were dichotomized as “step performed correctly” (=1); or “step executed incorrectly” (=0). Furthermore, a total score was calculated per person.

##### Phase 2: Satisfaction and acceptability

The scores for the 10-point likert scale assessing difficulty of use were expressed as medians, ranges and interquartile ranges (IQR) for all participants, volunteers, and patients.

Qualitative analyses of the interview transcripts were performed by two researchers independently. For thematic deductive content analysis [20], meaningful comments were identified regarding the Helping Hand™’s usability aspects, then defined according to the framework for testing electronic adherence monitoring devices (De Bleser *et al.*, work in progress). After this, relevant quotations were grouped into the usability categories described in this study’s conceptual framework (De Bleser *et al.*, work in progress). This step required the construction of data matrices [21], the horizontal axis of which contained the subcategories of user performance, satisfaction, and acceptability. Within the ‘user environment’ sub-dimension, analysis of users’ comments led to the subcategories of ‘sound’, ‘light’ and ‘dust/heat’. The vertical axis included subjects’ scores, differentiating patients’ from those of volunteers.

In the next step, where possible, each of the usability subcategories were recoded, e.g., quotations concerning the feedback function within the ‘medical device user interface characteristics’ sub-dimension were recoded as ‘problems with feedback function’, ‘no problems with feedback function’ or ‘no opinion on this topic’. Recoding was done independently by two researchers. The initial agreement among the two researchers was 85.4%. For quotations for which no agreement was found, meetings with a third researcher were held until consensus was reached. To quantify the qualitative data [22], the number of subjects citing each subcategory was specified.

All phases of the project were reviewed by an external auditor (MBH) based in the USA who has experience in qualitative and mixed-method research. All interviews and questionnaires were in Dutch. After the analysis they were translated into English to allow communication within the research team [23].

## 4. Results

Overall, 21 individuals participated, of which 11 were KTx patients and 10 healthy volunteers. Subjects’ demographic characteristics are summarized in Table 1.

Transcripts of the 21 interviews (both baseline and after 3-weeks) revealed 178 relevant quotations (90 from patients, 88 from healthy volunteers). These were categorized broadly as ‘performance’, ‘satisfaction’ and ‘acceptability’.

## 5. User Performance (Phase 1)

### Method I: Number of Helping Hand™ user errors

#### 

Operating the HH entails 20 critical steps: 5 for activation; 7 for reprogramming; and 8 daily use of the device. We have evaluated the number of errors for each of these 20 steps (Table 2). Fifty-seven percent (12/21) of subjects used the HH without error. If errors occurred, they were mostly related to users not hearing the device’s sound signal, e.g., almost 18% of the patients and 40% of the healthy volunteers heard nothing when inserting the blister card (Table 2A). Inserting blister cards was also problematic: 28% of the patients and 20% of the volunteers were unable to insert the blister card into the Helping Hand™. Reprogramming (Table 2B) the device was performed faultlessly by 48% of the subjects. However, 45% of the patients and 10% of the volunteers made blister card-related errors (*i.e.*, they moved their cards too slowly to begin reprogramming or not far enough into the device to signal use). Most frequently occurring error in the daily use of the HH by patients (Table 2C) were related with reinserting the tablet card into the Helping Hand™.

We asked whether the device was appropriate for specific patient groups (Table 3B). Six (28.7%) subjects (4 patients and 2 healthy volunteers) answered that the reminder function could be particularly helpful for elderly people, however, an equal number suggested that elderly persons would have trouble removing and re-inserting the medication blister cards. Six patients (29%) also saw applicability among recent transplant recipients.

##### Medical device user interface characteristics

Three (27%) patients and 2 (20%) volunteers reported confusion with the device’s three feedback lights (Table 3C). Four patients (36%) and 7 (70%) volunteers cited difficulty inserting and removing the blister cards. Once several pills had been removed, cards began to curve, making reinsertion into the device difficult.

Quote 3: *‘Hoo-ha, it is not easy to re-insert the blister into the device, especially not when the card is slightly rumpled.’*

Apart from these issues, participants in general reported no problems with programming or using the Helping Hand™. Notably, on the questionnaire, six (55%) patients and 2 volunteers (20%) categorized it as ‘very time consuming’, but no participant mentioned this during the interview. In addition, 6 (54%) patients and 6 (60%) volunteers considered it to be a design weakness that the device was usable with only one medication.

Quote 4: *‘Nevertheless, it is hard that only one… [medication] can be inserted, because, when I come home in the evening, I still have to take the others or take along the others.’*

## 6. Satisfaction (Phase II)

The overall range of perceived satisfaction was very broad. On a 5-point Likert scale (Table 4), one (9%) patient and 1 (10%) volunteer scored ‘not satisfied at all’ while 2 (18%) and 2 (20%) volunteers were very satisfied. Regarding perceived helpfulness, 40% of patients found the device ‘very helpful’, while another 40% found it ‘not helpful at all’. (Table 4) 2 (18%) patients and 2 (20%) volunteers were very positive, while one (9%) patient and 1 (10%) volunteer were very negative (Table 4).

### 6.1. Physical dimension

Opinions on the size and shape of the device varied widely. After the 3-week monitoring period, roughly half of patients and volunteers mentioned that the device was too large and that its curved shape was rather ‘strange’. All participants considered the reminder sound too quiet. Five (50%) volunteers reported that the light signal was difficult to see in bright ambient light.

Quote 5: *‘That sound signal is a total fiasco. If that is not adapted, it will be classified as a toy.’*

### 6.2. Privacy dimension

Nearly a quarter of the sample (2 patients and 3 volunteers) expressed doubts about the device’s *privacy aspect,* mainly because the pills were visible inside the device (Table 5).

#### 

##### Human interaction dimension

Most patients appreciated the device’s monitoring function, *i.e.*, that the patterns of medication intake are downloadable by clinicians and can be shown for feedback to patients (Table 5). Only two patients (18%) stated that they would not want their physicians to ‘control’ them.

Quote 6: ‘I would like to show my physicians the results, so they can see that you are doing well.’

### 6.3. Self concept dimension

Nine (82%) patients and 8 (80%) volunteers mentioned that the device had a positive influence on their *self concept*, helping them to take their medication more strictly (Table 5).

Quote 7: ‘Before, I didn’t, I must be honest. In the morning it was always correct because I woke up at approximately the same time. In the evening, I took them later. But now, during the three weeks, I have paid more attention to it.’

### 6.4. Routine dimension

Seven patients (64%) and two volunteers (20%) mentioned that using the Helping Hand™ in combination with a pillbox was not problematic for them (Table 5).

### 6.5. Sustainability dimension

Three (27%) patients and 1 (10%) volunteer expressed feelings of dependency on the device as a reminder. On the other hand, 7 (64%) patients and 6 (60%) volunteers said they had no such feelings (Table 5).

Quote 8: ‘I don’t want to feel myself dependent on devices, unless it is necessary.’

## 7. Acceptability (Phase II)

Four (36%) patients and 4 (40%) volunteers said they would like to use the Helping Hand™ in the future. An equal number said they would use it after certain improvements, e.g., increasing the sound volume (Table 6). On the other hand, three (27%) patients and 1 (10%) volunteer ruled out ever using it again. Only 4 (36%) patients and 6 (60%) volunteers would recommend the device to others.

## 8. Discussions

This study evaluated the usability of the new Helping Hand™ electronic medication monitoring system. In general, this device’s goal was viewed positively by all subjects. The following paragraphs will outline the HH’s primary obstacles, along with its clinical implications. Finally, the methodological considerations of this study will be discussed.

### 8.1. User performance

This aspect of usability was measured in terms of the errors made while operating the HH; the time required to perform each step of the operation; and the interaction of subjects with the HH using the think-aloud method. Errors were observed in 0 to 45% of the respondents, and occurred mainly in the activation and reprogramming of the device. The time required to perform each operational step was acceptable. The think-aloud technique revealed the following problems: a weak sound signal; difficulties loading the medication; and the fact that only one medication could be used at a time.

Indeed, the device’s sound signal, which is important both as the medication reminder and as feedback during activation and reprogramming, was inaudible to many subjects. The tone used has a frequency of 2,048 Hertz. The human ear can detect frequencies between 30 Hz and 20,000 Hz, but the capacity to hear high frequencies decreases with age [24]. Nevertheless, even younger subjects could not hear the sound. Considering these aspects, it is recommended to use a lower frequency tone to increase audibility.

A second frequently mentioned obstacle was inserting the blister card into the device. This requires concentration and refined motor skills. In KTx patients, this can be a problem because some immunosuppressive drugs have tremor as side effect [25]. However, we observed that healthy volunteers with unimpaired fine motor skills also found card insertion difficult.

A third concern, specifically observed in KTx patients, was that only one medication blister could be inserted into the Helping Hand™. This is a limitation for patients on polypharmacy. This confirms previous findings, which showed that patients taking more than six different drugs daily evaluated the Helping Hand™ as less useful [26]. Rather than an operational issue, this could be considered as a limitation of the device.

### 8.2. Satisfaction

In general, this device’s goal was viewed positively by all subjects. The respondents found the HH easy to use, the time required to operate was acceptable, and they liked the device. They did not feel controlled by the device, they were not psychologically dependent on the device, and it did not interfere with their routine medication habits. In a previous study, Santschi [27] asked patients with hypertension for their opinions of two electronic monitoring systems. Only ten percent felt conscious of being observed, experiencing this as negative. Yet, subjects who participated in Santschi’s study already had few problems with adherence and therefore did not mind ‘being observed’. In addition, the Helping Hand™ can facilitate communication between patient and physician on medication adherence, and can be therefore seen as a supportive tool. [26]

On the other hand, respondents did report some remarks concerning the device. They experienced the sound as to weak; and felt that the size of the device was too big. Indeed, the size was a matter of some concern, as users generally considered it important to conceal the device. As previous studies have also shown, subjects attached considerable importance to the monitoring system’s compactness and portability [27–30]. In general, the respondents questioned the usefulness of this device, except for newly transplanted patients. For instance, patients who had not yet developed stable medication taking routines could benefit from a reliable reminder. Wildin [31] supports these results, showing that patients already managing their own medication behavior stably are extremely unlikely to adopt new medication adherence aids. Bova *et al.* also suggested that patients already using pillboxes would avoid the use of an electronic monitoring system [32,33].

### 8.3. Acceptability

Respondents found the use of the HH acceptable, but technical improvements are required. About half of the respondents would recommend the use of this device to others. Differences were however found between healthy subjects and KTx patients. Healthy individuals would be prepared to pay less, on average, than patients for the device.

### 8.4. Methodological issues

We experienced some methodological issues during the execution of this study. First, the think aloud method, which allows/requires the user to log his/her thoughts and actions during their operation [11], was difficult to teach. To make participants familiar with this method, we trained them by employing the think aloud method while making a puzzle, or by explaining how to make tea.

Second, the subjects were unfamiliar to the Helping Hand™. Therefore, they required training in using the device. After the training session, the respondents were asked to demonstrate adequate management of the device. However, the researcher had to intervene occasionally when subjects encountered insurmountable problems while executing an order. It is assumed that the real training process of the device probably happened after the researcher has left, when the subject had to reprogram the device for the first time without assistance.

Third, it is suggested that large-scale samples are redundant in user experiences research. Nielsen [34] asserts that reliable results can be obtained from samples of five subjects [34]. However, in our study, following the more widely accepted assumption that 8 to 12 people are necessary in qualitative research [35], we aimed for 12 volunteers and 12 patients. In fact, we suspected that data saturation would be achieved well before the end of the interview process, which was eventually the case.

Fourth, subject answers on satisfaction and acceptability could be influenced by several confounding factors. One possible confounder is that socially desirable responses were given on the questionnaire, which was completed by the respondent in the researcher’s presence. To minimize this effect, subjects were assured that the study was purely descriptive, that no wrong answers were possible, and that their medication behavior is not being evaluated. It was also emphasized that all information was totally confidential. Furthermore, to avoid forcing judgments, each questionnaire item included an option to express ‘no opinion’.

### 8.5. Practical implications

Before using the device for a sustained period, subjects would like to see adaptations of certain design aspects of the helping Hand™. The weak sound signal, the narrow blister card loading slot, and the fact that only one medication blister at the time can be used should be changed before the HH can be used routinely in clinical practice and/or daily life.

### 8.6. Future research

Although this study provides information to improve the usability of the HH, further research is needed. Future studies can replicate our methods after the technical improvements have resolved the main obstacles. However, it could be relevant to conduct future studies in a usability lab. Such a lab comprises a closed video circuit that records users while interacting with the device. Moreover, it could be relevant to undertake such studies in other patient populations to take specific patients characteristics, such poor vision, audition and motor function, into account.

## 9. Conclusions

This study evaluated the usability of the new Helping Hand™ electronic medication monitoring system. The device’s intended use—facilitating, reinforcing and monitoring a medication routine—was received positively by all subjects. Likewise, the device’s reporting function, which allows physicians to review users’ medication behavior, was scored positively by all patients except two. However, the feedback system proved to be confusing. In general, the performance of the device did not meet subjects’ expectations. The most important obstacles to operation were the weak sound signal, problems loading the medication, and the fact that only one medication could be used at a time.

## Figures and Tables

**Table 1.** Demographic characteristics of the sample.

	**Patients n = 11 (%)**	**Healthy volunteers n = 10 (%)**

Median age, (Q1–Q3) (range)	62 (54–71) (25–74)	56 (35–72) (28–80)

Gender		
Male	6 (54.5)	4 (40)
Female	5 (45.5)	6 (60)

Educational level		
Low (<15y of age at finishing education)	6 (54.5)	2 (20)
Moderate (<18y of age at finishing education)	2 (18.2)	4 (40)
High (>18y of age at finishing education),	3 (27.3)	4 (40)

Work situation		
Fulltime	3 (27.3)	4 (40)
Part time	0	1 (10)
Retired	6 (54.5)	3 (30)
Housewife	1 (9.1)	1 (10)
Incapacity to work,	1 (9.1)	1 (10)

Marital status		
Single	1 (9.1)	1 (10)
Married/living together	9 (81.2)	9 (90)
Divorced	0	0
Widowed	1 (9.1)	0

Median time after Tx (years), (Q1–Q3) (range)	9 (3–13) (2–16)	/

**Table 2.** Number of mistakes during the use of the Helping Hand™.

		**Patients (n = 11)**	**Volunteers (n = 10)**
**2 A. Number of mistakes during the activation of the Helping Hand™**			
1	Choose the point of time you want starting the alarm system	0	0
2	Insert the tablet card	3 (28%)	2 (20%)
3	Hear one beep tone	2 (18%)	4 (40%)
4	See the light is blinking, red, orange and green in that order	2 (18%)	0
5	See the green light only is blinking	1 (9%)	0
**2 B. Number of mistakes during the reprogramming of the Helping Hand™**			
1	Move the blister in and out the device about 8 times	5 (45%)	1 (10%)
2	Hear the Helping Hand™ buzzing on movement no. 5 to 7, warning that the reset is about to take place	1 (9%)	3 (30%)
3	Hear 3 beep tones	1 (9%)	3 (30%)
4	Be aware that the 3 tones confirm that the reset has been performed	0	2 (20%)
5	Start up the Helping Hand™ by inserting the blister again into the device	4 (36%)	0
6	Be aware that the reset is not performed if the in/out sequence is interrupted. The reminder of the Helping Hand™ starts beeping at the original time	NA	NA
7	Be aware that the device is now ready to be used	NA	NA
**2 C. Number of mistakes the daily use of the Helping Hand™**			
1	Be aware that the Helping Hand™ starts beeping when you should take your medication	NA	NA
2	Be aware that, at the same time, the **red** light starts flashing	NA	NA
3	Remove the tablet card out of the Helping Hand™	0	0
4	Take your medication as prescribed by the treating physician	0	0
5	Hear the Helping Hand™ beeping once	0	3 (30%)
6	Re-insert the tablet card	3 (28%)	0
7	See the light flashing	2 (18%)	0
8	Be aware that the colour of the light flashing depends on the number of doses missed	0	1 (10%)

NA: Not applicable because action did not take place at that moment

**Table 3.** Results on performance of the Helping Hand™ (patients: n = 11, volunteers: n = 10).

**Performance**				
**A. Use environment (physical load)**		No problem (%)	Problem (%)	No answer (%)

Light	Patients	0 (0)	4 (36)	7 (64)

	Volunteers	0 (0)	0 (0)	10 (100)

Sound	Patients	0 (0)	3 (27)	8 (80)

	Volunteers	0 (0)	0 (0)	10 (100)

Dust/heat	Patients	0 (0)	2 (18)	9 (82)

	Volunteers	0 (0)	0 (0)	10 (100)

**B. User characteristics**		Appropriate for (%)	Not appropriate for (%)	No answer (%)

People with sensory capacity problems	Patients	0 (0)	1 (9)	10 (91)

	Volunteers	0 (0)	5 (50)	5 (50)

People with cognitive capacities & memory problems	Patients	4 (36)	0 (0)	7 (64)

	Volunteers	4 (40)	1 (10)	5 (50)

Elderly people	Patients	4 (36)	2 (18)	5 (45)

	Volunteers	2 (20)	4 (40)	4 (40)

Recently transplanted patients	Patients	6 (54)	0 (0)	5 (45)

	Volunteers	0 (0)	0 (0)	10 (100)

For people being at home all day	Patients	4 (36)	0 (0)	7 (64)

	Volunteers	0 (0)	0 (0)	10 (100)

**C. Medical device user interface characteristics**		No problem (%)	Problem (%)	No answer (%)

Interpretation of the feedback function	Patients	6 (54)	3 (27)	2 (18)

	Volunteers	5 (50)	2 (20)	3 (30)

Blister movement	Patients	6 (54)	4 (36)	1 (9)

	Volunteers	2 (20)	7 (70)	1 (10)

Use of HH in general	Patients	4 (36)	2 (18)	5 (45)

	Volunteers	3 (30)	2 (20)	5 (50)

Programming	Patients	9 (82)	2 (18)	0 (0)

	Volunteers	8 (80)	2 (20)	0 (0)

Amount of time consumed while using the HH	Patients	5 (45)	0 (0)	6 (54)

	Volunteers	8 (80)	0 (0)	2 (20)

Only 1 medication can be inserted	Patients	0 (0)	6 (54)	5 (45)

	Volunteers	0 (0)	6 (60)	4 (40)

**Table 4.** Likert scales on the satisfaction and the acceptability of the Helping Hand™ (patients: n = 11, volunteers: n = 10).

**Score on Likert scale**		**1 Not at all…**	**2**	**3**	**4**	**5 Very…**
How easy was it to use the device?	Patients	1 (9%)	2 (18%)	0 (0%)	1 (9%)	7 (64%)
	Volunteers	0 (0%)	1 (10%)	0 (0%)	3 (30%)	6 (60%)
How enjoyable was the device to use?	Patients	2 (18%)	1 (9%)	6 (55%)	1 (9%)	1 (9%)
	Volunteers	2 (20%)	1 (10%)	4 (40%)	2 (20%)	1 (10%)
How helpful was using the device for you?	Patients	2 (18%)	4 (40%)	3 (27%)	1 (9%)	1 (9%)
	Volunteers	4 (40%)	0 (0%)	1 (10%)	1 (10%)	4 (40%)
How much did you like the device?	Patients	2 (18%)	0 (0%)	0 (0%)	3 (27%)	6 (55%)
	Volunteers	0 (0%)	0 (0%)	1 (10%)	2 (20%)	7 (70%)
How acceptable was the time you needed for using the device?	Patients	2 (18%)	0 (0%)	0 (0%)	3 (27%)	6 (55%)
	Volunteers	1 (10%)	1 (10%)	3 (30%)	3 (30%)	2 (20%)
Overall, how satisfied are you with the device?	Patients	1 (9%)	1 (9%)	4 (40%)	3 (27%)	2 (18%)
	Volunteers	1 (10%)	1 (10%)	3 (30%)	3 (30%)	2 (20%)

**Table 5.** Results on satisfaction of the Helping Hand™ (patients: n = 11, volunteers: n = 10).

**Satisfaction**				
**Physical dimension**		No problem (%)	Problem (%)	No answer (%)

Size	Patients	6 (55)	5 (45)	0 (0)

	Volunteers	2 (20)	6 (60)	2 (20)

Shape	Patients	5 (45)	4 (36)	2 (18)

	Volunteers	5 (50)	5 (50)	0 (0)

Color	Patients	4 (36)	0 (0)	7 (64)

	Volunteers	4 (40)	1 (10)	5 (50)

Sound	Patients	0 (0)	11 (100)	0 (0)

	Volunteers	0 (0)	10 (100)	0 (0)

Light signal	Patients	5 (45)	0 (0)	6 (55)

	Volunteers	1 (10)	5 (50)	4 (40)

**Privacy dimension**	Patients	8 (73)	2 (18)	1 (9)

	Volunteers	5 (50)	3 (30)	2 (20)

**Human interaction dimension** (control by physician)	Patients	6 (55)	2 (18)	3 (27)

	Volunteers	8 (80)	0 (0)	2 (20)

**Self-concept interaction dimension** (positive influence)	Patients	9 (82)	2 (18)	0 (0)

	Volunteers	8 (80)	2 (20)	0 (0)

**Routine dimension** (used in combination with pill box)	Patients	7 (64)	0 (0)	4 (36)

	Volunteers	2 (20)	0 (0)	8 (80)

**Sustainability dimension** (feeling dependent from the device)	Patients	7 (64)	3 (27)	1 (9)

	Volunteers	6 (60)	1 (10)	3 (30)

**Table 6.** Results on the acceptability of the Helping Hand™ (patients: n = 11, volunteers: n = 10).

**Acceptability**					
		Yes (%)	No (%)	Yes, if technical improvements are made (%)	No answer (%)
Acceptable to use HH in the future	Patients	4 (36)	3 (27)	4 (36)	0 (0)
	Volunteers	4 (40)	1 (10)	4 (40)	1 (10)
Recommend it to others	Patients	4 (36)	2 (18)	0 (0)	5 (45)
	Volunteers	6 (60)	1 (10)	0 (0)	3 (30)
